# Consequences of a Delayed Diagnosis of Kaposi’s Sarcoma: A Case Report of Disseminated Infection

**DOI:** 10.3390/idr13010017

**Published:** 2021-02-06

**Authors:** Leonardo Henrique Bertolucci, Carolina Rossatto Ribas, Ellen Mullich Flesch, Lisiane Aurélio Knebel Balbinot, Fabiano Ramos

**Affiliations:** 1 Medicine School, Pontifícia Universidade Católica do Rio Grande do Sul, Av. Ipiranga, 6681, Porto Alegre 90619-900, Brazil; ellen.flesch@acad.pucrs.br (E.M.F.); lisianeknebel@gmail.com (L.A.K.B.); 2 Hospital São Lucas da PUCRS, Pontifícia Universidade Católica do Rio Grande do Sul, Av. Ipiranga, 6681, Porto Alegre 90619-900, Brazil; carolina-rribas@hotmail.com (C.R.R.); fabiano.infecto@gmail.com (F.R.)

**Keywords:** Kaposi’s Sarcoma, HIV, HHV-8, AIDS, KS

## 1. Introduction

Kaposi’s Sarcoma (KS), first reported by Dr. Moritz Kaposi in 1872, is the most common vascular tumor in patients with AIDS. This disease is characterized by the appearance of violet lesions in the skin, mucosa, and gastrointestinal tract and may also affect other organs. KS is a multicentric angioproliferative spindle cell tumor originating from human herpes virus type 8 (HHV-8)-infected endothelial cells from lymphoid tissue [1].

Most patients have painless skin lesions, which may have macular, papular, nodular, or plaque appearance. The lesions can have a range of colors from pink to red or purple and range in size from several millimeters to large confluent areas. Lesions are typically located in the oral cavity, face, and lower extremities, but may involve other sites. Visceral disease sometimes occurs in the absence of cutaneous lesions. Oral lesions can lead to ulceration, dysphagia, and secondary infection [2].

In the immunocompetent host, the clinical course of HHV-8 infection is generally asymptomatic. In immunocompromised hosts, such as post-transplant patients and HIV-infected patients, HHV-8 develops into a variety of lymphoproliferative disorders [1].

While HHV-8 is highly seroprevalent in sub-Saharan Africa (>50%), it is quite rare in most European countries, Asia, and the United States (<10%) [3]. The seroprevalence of HHV-8 is high in men who have sex with men [4] and its transmission occurs through saliva and through sexual intercourse [5,6].

There are currently five clinical variants of KS that have been described:

1. Classical [7]: cutaneous/indolent form in elderly Mediterranean European men.
2. Endemic [7]: An aggressive form of non-AIDS KS, usually seen in sub-Saharan Africa, usually with visceral involvement.
3. Iatrogenic [7]: related to solid organ transplantation and patients receiving immunosuppressive drugs.
4. Epidemic/associated with acquired human immunodeficiency virus (HIV) infection [7]: more frequent in young adult gay and bisexual men.
5. Non-epidemic Kaposi [8]: fifth and most recent, described in non-HIV-positive patients but at high risk for HIV.

KS associated with AIDS exhibits a broad spectrum of clinical presentations. The prognosis relies on the stage of KS, the level of immunosuppression, and the response to highly active antiretroviral therapy (HAART) [1].

HIV-induced immunosuppression is a crucial factor in inducing the development of KS. The decrease in absolute CD4 T cell counts and the decrease in HHV-8-specific T cell immunity are associated with KS [9]. In addition, the development of KS is independently associated with the degree of HIV viremia [10].

The initial treatment for AIDS-related KS is with HAART [11], which, from the beginning of its introduction, has proven effective for controlling this sarcoma. Treatment strategies include local or systemic approaches. Among the methods of effective local intervention in the control of cutaneous KS lesions are cryosurgery or laser therapy, cytoreductive surgery, therapy with immunomodulatory agents applied topically, and intralesional chemotherapy, in addition to retinoid agents [12]. However, when this neoplasm is at a later stage, it is necessary to include additional therapies for treatment such as chemotherapy (method of choice for the patient reported in addition to antiretroviral therapy), radiotherapy, and immunotherapy. Therefore, the importance of early diagnosis is essential, since it guarantees the best prognosis for the patient.

## 2. Case Report

A male, 35 years old, with no known comorbidities was admitted to the emergency department, referred by the Basic Health Unit, due to edema and non-painful violet lesions in lower limbs without clear etiology, in addition to losing more than 10 kg in 2 months.

**Figure 1:**
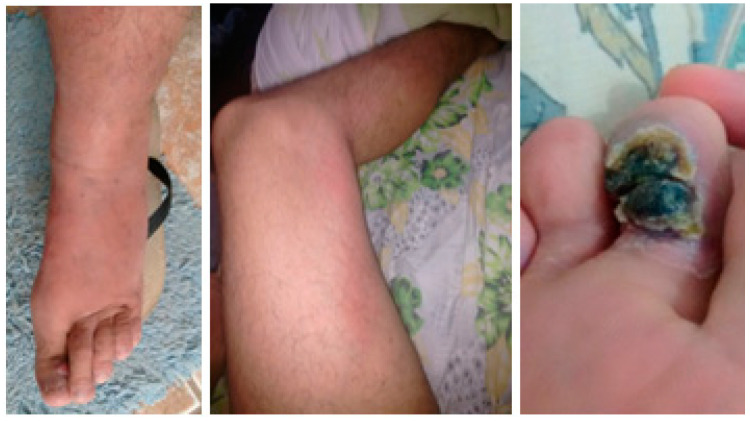
Initial presentation of verrucous lesions, erythematous cord, and edema. These pictures were taken by the patient 12 months before looking for an emergency.

The patient reported that the clinical picture started 12 months ago, initially with edema of the left lower limb, accompanied by a longitudinal erythematous cord that extended from the thigh to the ipsilateral foot, in addition to a single verruciform lesion located in the plantar region of the second toe (Figure 1). The patient reported that he visited several hospitals and medical centers, in which he made use of antimicrobial treatments, with no resolution of the condition. In addition, he informed that the disease progressed in these 12 months, stating that the verruciform lesions started to affect the other toes of the left foot, and later, the involvement was similar in the right lower limb, with bilateral lesions and edema at that moment generating pain and important limitation to ambulation. He also reported that he has sexual intercourse with men, that for about 2 months, he had no appetite, and that 3 years, ago he had herpes zoster.

**Figure 2:**
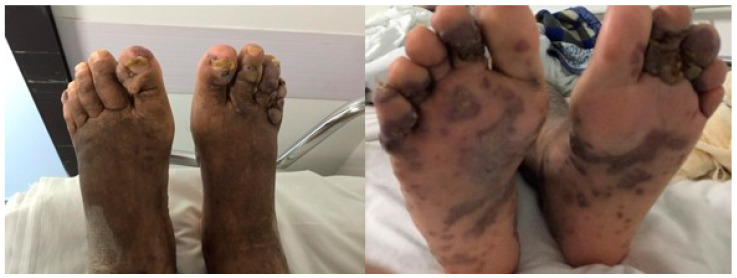
Disseminated Kaposi’s Sarcoma with Cutaneous Verruciform Kaposi in feet.

On physical examination, the patient presented with pale mucous membranes and was emaciated. Upon inspection of the lower limbs, edema 4+/4+ without locker, in addition to evident hyperchromic lesions and hyperpigmented plaques with irregular edges and verruciform lesions, especially in the toes of both feet (Figure 2). In addition, there were similar violaceous lesions on the right forearm, with a single lesion on the back. In the oral cavity, he had oral candidiasis.

Laboratory examinations of the patient’s arrival were performed. It was observed that the patient had anemia and leukopenia with lymphocytopenia. In addition, he was diagnosed with HIV on his arrival at the hospital. He had a CD4 of 31 cells/µL, a CD8 of 301 cells/µL, a CD4/CD8 ratio of 0.10, and a viral load of 474,071 copies. Skin biopsies of the lesions were performed, which resulted in a suggestive aspect of KS. 

**Figure 3:**
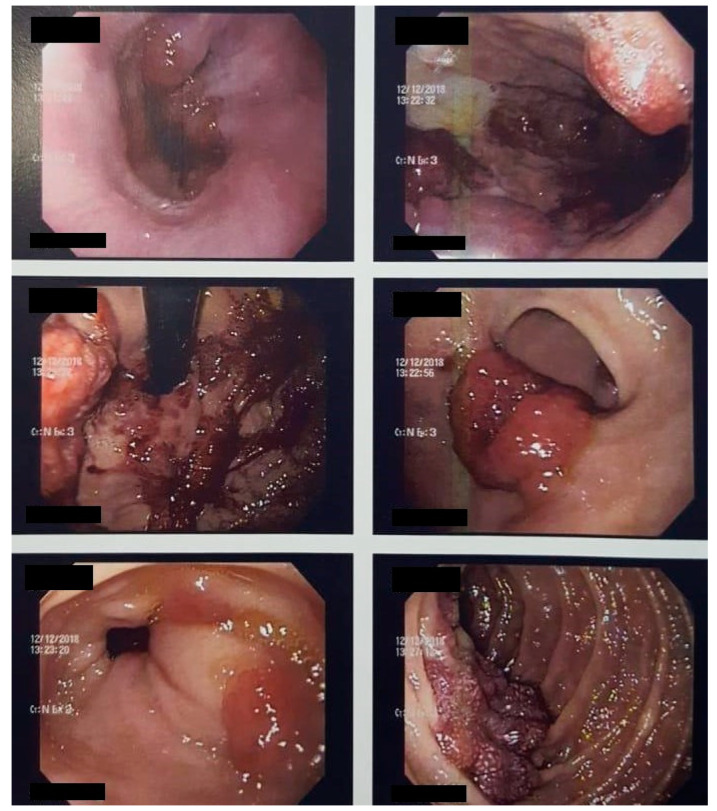
Upper digestive endoscopy identified the presence of diffuse violet lesions of nodular forms, not determining obstruction, at the cardia, body, fundus, and antrum.

Computed tomography (CT) scan of the chest indicated numerous irregular opacities with ill-defined limits accompanied by small peribroncovascular pulmonary nodules, located in both lungs. The abdominal CT scan showed a liver with usual contours and dimensions, observing two small hypoattenuating images in the right lobe, both measuring 0.7 cm, undetermined. Fibrobronchoscopy revealed a trachea with two violet spots on the anterior wall which were biopsied, resulting in a lesion suggestive of KS. Upper digestive endoscopy identified the stomach with the presence of diffuse violet lesions of nodular shapes, without determining obstruction (Figure 3). In colonoscopy, a device was introduced up to the hepatic angle, where there was an injury that prevented the passage of the device. In all colonic segments examined was the presence of multiple elevated lesions, with lobulated and violet appearance. Biopsy lesions of the gastrointestinal tract indicated KS.

After the diagnosis, the patient started follow-up with the Infectious Diseases and Oncology Departments, starting HAART and liposomal doxorubicin as a treatment for KS.

## 3. Discussion

Considering that the patient did not initially present the characteristic violaceous lesions of KS, several erroneous diagnoses were made during the 12 months of the beginning of the first symptoms, and numerous wrong treatments were prescribed to the patient, contributing to the delay in his diagnosis and impairment of the patient’s prognosis. When the patient was admitted, the lesions were already presented in a typical form, with violet coloration. In addition, the oral cavity, trachea, lungs, stomach, and colon had already been affected.

## 4. Conclusions

The main interest of this report is to emphasize the importance of early diagnosis. Despite the fact that the incidence of KS is decreasing with the emergence of HAART, this neoplasm still can indicate an unknown HIV status and significantly alter the prognosis and the survival of patients as the diagnosis is delayed.

## Data Availability

No new data were created or analyzed in this study. Data sharing is not applicable to this article.
